# Recent advances in the management of transient ischemic attacks

**DOI:** 10.12703/r/11-19

**Published:** 2022-07-22

**Authors:** Jorge Ortiz-Garcia, Camilo R Gomez, Michael J Schneck, José Biller

**Affiliations:** 1 Department of Neurology, The University of Oklahoma Health Sciences Center, Oklahoma City, OK, USA; 2 Department of Neurology, University of Missouri Columbia, Columbia, MO, USA; 3 Department of Neurology, Loyola University Chicago, Stritch School of Medicine, Maywood, IL, USA

**Keywords:** transient ischemic attack, TIA, stroke risk stratification, antiplatelet therapy, anticoagulants, arterial revascularization

## Abstract

Transient ischemic attack (TIA) constitutes an important clinical condition, indicating the presence of considerable risk for a subsequent ischemic stroke. Its prompt diagnosis and management have the potential for reducing the risk of neurologic disability, highlighting the critical need to prioritize the care of patients with TIA. The risk of ischemic stroke following a TIA is directly related to its etiopathogenesis, and recognizable causes are commonly categorized within one of three domains: cerebrovascular pathology, cardiac dysfunction, and hematologic disorders. Therefore, the clinical approach to patients suspected of having suffered a TIA demands a comprehensive evaluation, including testing of possible etiologic conditions in all three of these domains, best carried out in an expedited fashion since the stroke risk is greatest in the hours and days that follow the index event. The present is a review of the existing literature addressing the diagnosis, evaluation, prioritization, and management strategies available to clinicians who provide care to patients with TIA.

## Introduction

The diagnosis of transient ischemic attacks (TIAs) is essential to reduce the risk of subsequent ischemic stroke; this risk can be as high as 20% in the 3 months following TIA depending on the causative mechanism of the index cerebrovascular event^1^. Therefore, the goals for evaluation and management of patients suspected of having a TIA are to establish a clinical diagnosis with as much certainty as possible, define the etiologic mechanism of the TIA, risk-stratify the patient for possible subsequent stroke, and implement a management plan to prevent the recurrent event. This review focuses on implementing these goals effectively, emphasizing the application of the recent literature in the decision-making processes.

## Historical overview

The term “transient ischemic attack” (TIA) was introduced at the Second Princeton Cerebrovascular Disease Conference in 1956 by C. Miller Fisher, who presented a detailed characterization of episodic cerebral ischemia that “may last from a few seconds up to several hours, the most common duration being a few seconds up to 5 or 10 minutes”^2^. On his review of the existing literature, Fisher described prior TIA cases by Peabody (1891), Russell (1909), and Osler (1911), who attributed pathogenesis of TIA to vasospasm^2^. Over time, the duration of TIA continued to be a matter of controversial opinion^3^. It would be nearly 20 years later, in 1975, that the National Institutes of Health formally classified TIAs as “episodes of temporary and focal cerebral dysfunction of vascular origin, rapid in onset (no symptoms to maximal symptoms in less than five minutes and usually less than a minute), commonly lasting 2 to 15 minutes but occasionally lasting as long as 24 hours”^4^, although, as previously noted by Fisher, the limit of 24 hours was arbitrarily chosen^2^.

**Figure 1.**
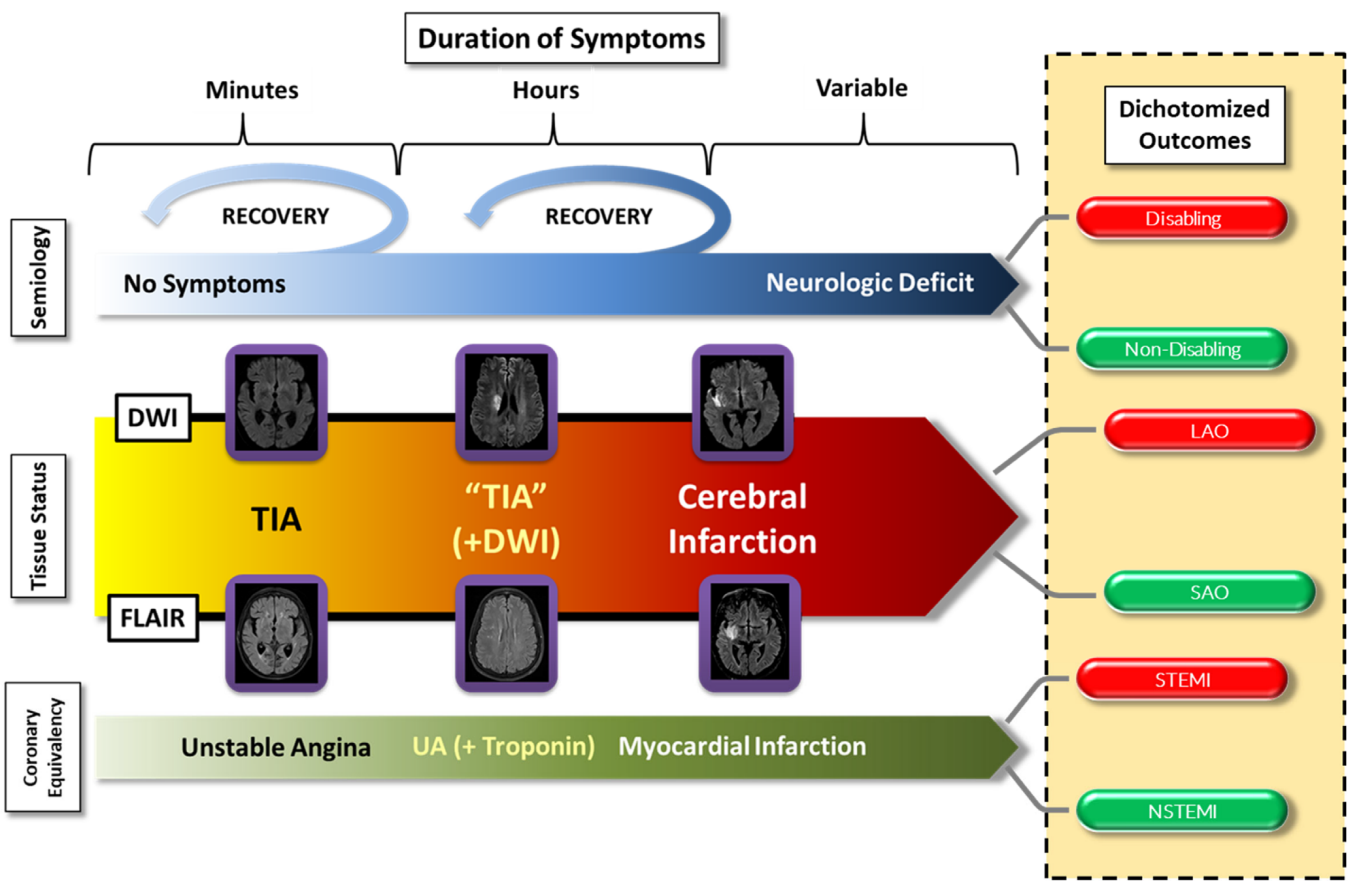
The continuum of acute cerebrovascular syndromes. The incorporation of both clinical aspects (i.e., specific syndrome and duration of symptoms) and the imaging status (i.e., imaging evidence of tissue injury or not) underscores the importance of considering transient ischemic attack (TIA) as part of a continuum rather than a discrete and separate entity from cerebral infarction. It also allows an overall understanding that is comparable to the one widely used for the coronary circulation. In this context, ischemic strokes due to large arterial occlusion (LAO) would be comparable to ST-segment elevation myocardial infarctions (STEMIs), while smaller infarctions would be more analogous to non-STEMIs (NSTEMIs). On another dimension, the presence or absence of a neurologic deficit may or may not be associated with evidence of tissue injury and, depending on the location and volume of the compromised brain tissue, may or may not result in a disabling outcome. See text for further description. DWI, diffusion-weighted imaging; FLAIR, fluid-attenuated inversion recovery; SAO, small arterial occlusion; UA, unstable angina.

Over the years, as advances in imaging technology were introduced, it became clear that a more precise conceptual approach to TIAs was necessary. Two important arguments drove TIA discussions: (a) with prolongation of symptoms (i.e., hours rather than minutes), the likelihood of a demonstrable ischemic lesion on imaging studies increased, and (b) even patients with demonstrable ischemic injury on imaging studies could have transitory clinical syndromes; based on these considerations, a formal recommendation for a more precise definition of TIA was advanced^5^ and subsequently adopted in revised American Heart Association/American Stroke Association guidelines. This definition specifies that the episode of transient neurologic dysfunction must be “caused by focal brain, spinal cord, or retinal ischemia, without acute infarction”, effectively shifting the emphasis from a time-based diagnosis to one that includes a tissue-based dimension^6–8^. This anatomo-clinical approach was a departure from the historical, phenomenological view to an approach rooted in a pathophysiologic construct supported by currently available imaging techniques. Our contemporary view of TIA fits into the continuum of *acute cerebrovascular syndromes*^9,10^, analogous to the paradigm of unstable angina among acute coronary artery syndromes, prioritizing the prevention of irreversible ischemic brain injury (i.e., infarction) (Figure 1). These similarities in cerebrovascular and coronary insufficiency extend to the point that ST-elevation myocardial infarction (STEMI) and cerebral large arterial occlusions are both emergently managed by endovascular therapies, while both non-STEMI (NSTEMI) and smaller ischemic cerebral arterial events are managed more conservatively (Figure 1).

## Clinical diagnosis of transient ischemic attacks

The difficulty in diagnosing TIA is that, by definition, most patients present *after the fact*, when symptoms have abated and there are no residual neurologic deficits. In this context, we emphasize the importance of pursuing a *presumptive* or *working* diagnosis rather than a *definite* diagnosis. Clinicians should maintain a low threshold for applying comprehensive cerebrovascular diagnostic protocols in patients with *presumptive* TIA because of its potential as a harbinger of ischemic stroke. The clinical presentation of TIA encompasses composites of one or more of the historically taught “warning signs of stroke”, including those mnemonics such as FAST (face, arm, speech, and time) or BE-FAST (balance, eyes, face, arm, speech, and time) used in public education campaigns, without discounting other less typical symptoms that may also result from focal cerebral ischemia^11–15^. Clinicians must obtain a thorough history of the index event, and the literature supports a few general considerations that serve as a practical blueprint for completing this task. Specifically, motor and language complaints have a greater predictive value for focal ischemic deficits than isolated sensory symptoms (i.e., clinical weight of evidence). Also, transient symptoms are more likely the result of focal ischemia if attributable to occlusion of a specific artery (i.e., physiologic plausibility). Moreover, an abrupt onset (i.e., acute temporal profile) has greater predictability for cerebrovascular diagnosis than insidious symptoms. Therefore, details about the index event must be sought in the context of these considerations to construct a presumptive TIA diagnosis with a reasonable degree of medical certainty (i.e., with a preponderance of evidence)^11^. Special mention must be made to transient monocular blindness (i.e., amaurosis fugax) as a presenting symptom since it carries a comparable significance for subsequent stroke risk even though the ischemic process affects the retinal circulation (i.e., branching from the ophthalmic artery, in turn branching from the internal carotid artery). Finally, clinicians must consider the possibility that “atypical” symptoms are also etiologically related to decreased perfusion of a specific cerebral vessel, fulfilling the criterion of physiologic plausibility. In particular, “confusion” and “dizziness” are symptoms that merit further discussion. Regarding the former, a certain proportion of patients who present with “confusion” may in fact suffer from language abnormality (i.e., aphasia) rather than the global cerebral dysfunction associated with an acute confusional state. Similarly, based on clinical differences, a complaint of “dizziness” may imply the lightheadedness produced by global cerebral hypoperfusion (i.e., pre-syncope). However, it could also indicate the presence of vertigo, a symptom potentially associated with vertebrobasilar ischemia^16,17^. In this context, it is immaterial if the vertigo is considered to represent central or peripheral labyrinthine dysfunction since the vertebrobasilar system provides the blood supply to both the labyrinth and its brainstem connections via the anterior inferior cerebellar artery and its branches.

Most patients with TIA will, by definition, have a normal neurologic examination. However, in the continuum of acute cerebrovascular syndromes, two subpopulations can present with subtle, persistent neurologic deficits: (a) patients with a more protracted symptom duration who may have evidence of diffusion-weighted imaging (DWI) magnetic resonance imaging (MRI) abnormalities and (b) patients with acute ischemic injury on fluid-attenuated inversion recovery (FLAIR) MRI sequence attributable to the presenting, though resolved, symptoms (Figure 1). The neurologic deficits of these patients are typically discreet and non-disabling. Again, an understanding of the continuum of acute cerebrovascular syndromes, with its anatomic, physiologic, and clinical correlations, remains the cornerstone of a TIA diagnostic evaluation (Figure 1).

## Comprehensive diagnostic evaluation

**Figure 2.**
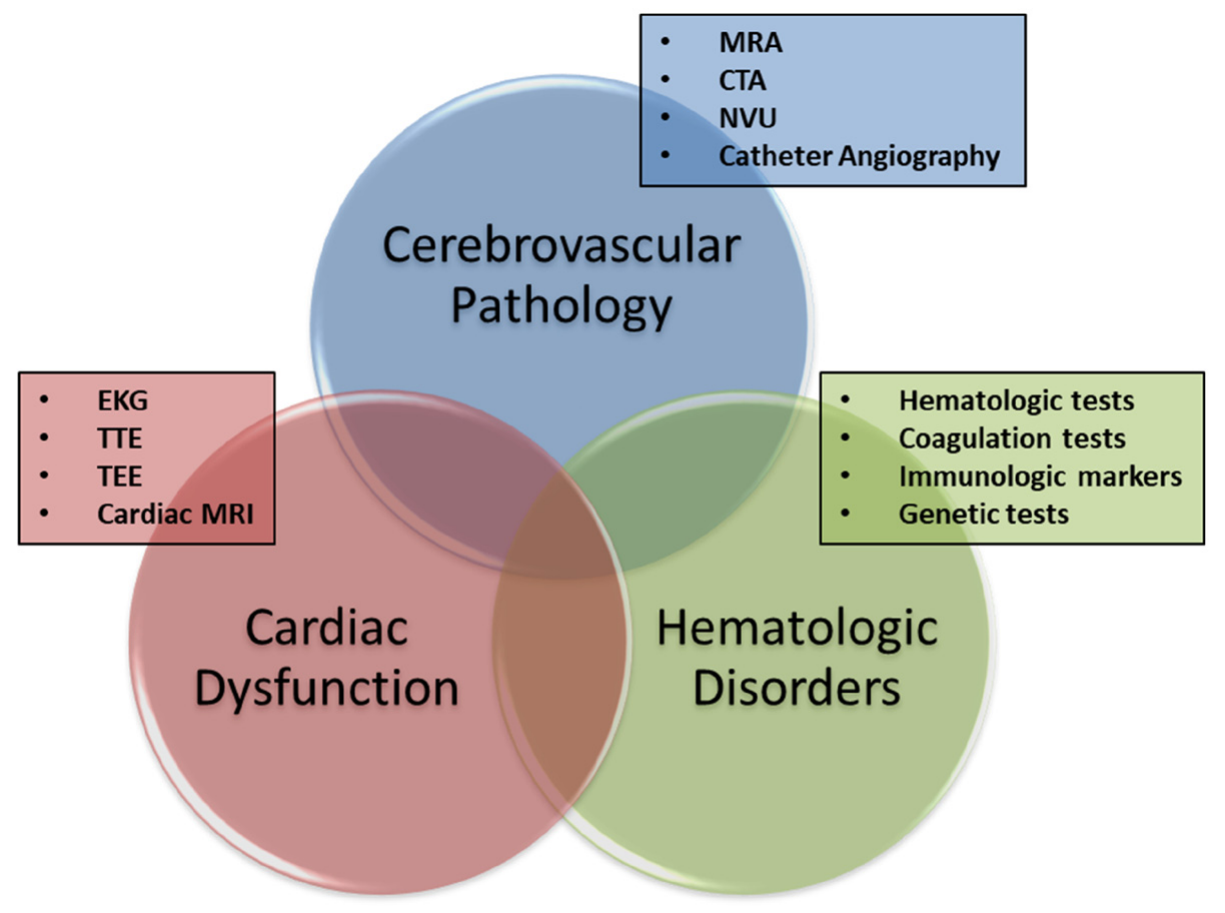
The three categories of causative disorders. All potential causes of transient ischemic attack can be allocated within one of three categorical concentric circles, and the areas of intersection constitute those patients with concurrent conditions (i.e., “double jeopardy”). Each of these three domains can be evaluated by means of different tests, listed in the figure. See text for further description. CTA, computed tomography angiography; EKG, electrocardiogram; MRA, magnetic resonance angiography; MRI, magnetic resonance imaging; NVU, neurovascular ultrasound; TEE, transesophageal echocardiogram; TTE, transthoracic echocardiogram.

Following a presumptive diagnosis of TIA, the next step is the determination of the index event’s pathogenesis in order to create a directed stroke risk reduction treatment strategy. As is the case in ischemic stroke evaluation, applying the principles of *Virchow’s triad* to the diagnostic evaluation of TIA leads to the investigation of possible cerebral arterial abnormalities (i.e., the “pipes”), cardiac dysfunction (i.e., the “pump”), and abnormalities of the blood contents (i.e., the “fluid”) (Figure 2). The practical application of these principles to a comprehensive TIA evaluation depends to a large extent on the clinical setting.

### Timing of the transient ischemic attack evaluation

As the primary objective of evaluating patients with TIA is prevention of subsequent ischemic stroke, clinicians should consider that entity a medical emergency and institute a quick and efficient diagnostic evaluation. The risk of stroke post-TIA varies widely, in accordance with the notion that TIAs result from very different causes, and the risk of a subsequent stroke depends largely on the underlying cause and mechanism of the index event^18–20^. Several scoring systems have been introduced through the years in an attempt to quantify the stroke risk of patients with TIA, and ABCD^2^ score is the most popular^21–25^. The ABCD^2^ score stratifies these patients into three different risk levels: low (<4), moderate (4–5), and high (6–7)^26,27^. However, low scores do not necessarily translate into minimal stroke risk, and about 20% of patients with an ABCD^2^ score of less than 4 have a 3-month stroke risk comparable to those with scores greater than 4^28,29^. While these scoring systems may have some aggregate value in stroke risk prediction, they do not include factors related to etiopathogenesis of the index TIA, the major driver of stroke risk. Thus, clinicians should not rely on TIA scoring systems to determine the urgency of the diagnostic evaluation^30–32^.

### Location of the transient ischemic attack evaluation

**Figure 3.**
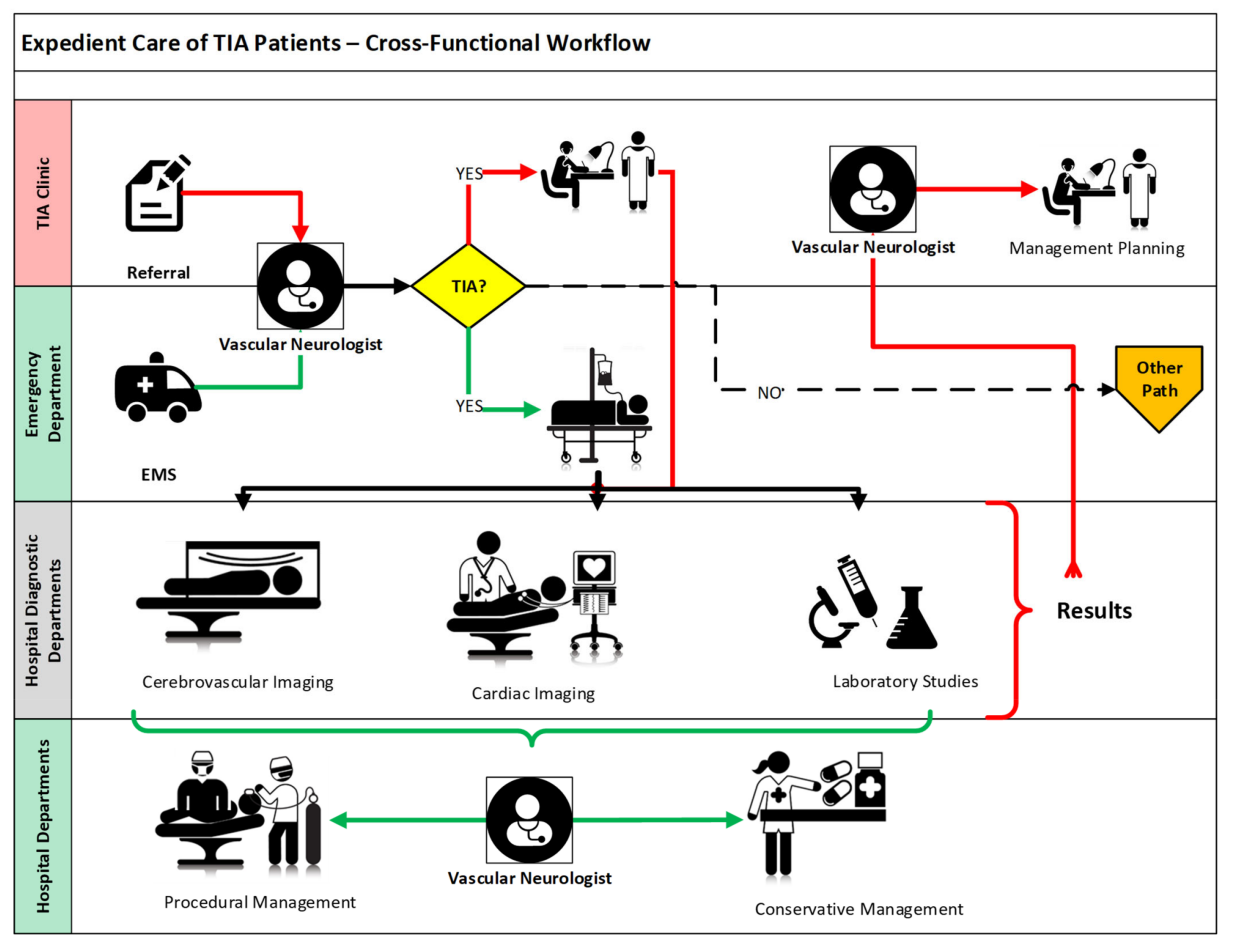
Cross-functional workflow for expedient care of patients with transient ischemic attack (TIA). Distinct sources for patient with TIA referrals are depicted in the two top swim lanes, leading to either an inpatient (green pathways) or an ambulatory (red pathways) evaluation. Segments common to both are depicted in black. Note how the inpatient evaluation pathway steadily moves in one direction (i.e., diagnosis, evaluation, then management), efficiently making use of the available physical resources in a sequential manner. Conversely, the ambulatory evaluation pathway requires that the patient be moved back and forth between the TIA clinic and the diagnostic facilities. The figure does not factor the time elapsed in completing either of these pathways, which is likely to be shorter for the inpatient evaluation. Note that the outpatient evaluation does not quite include management implementation, but just planning. EMS, emergency medical services.

Two opposing considerations apply regarding where and how to evaluate patients with TIA. Clinicians must balance the inconvenience and cost of hospitalizing patients with a normal neurologic examination with the potential delays associated with coordination of an extensive outpatient stroke evaluation and the possibility of worse functional outcomes. The optimal approach will vary based on the capabilities for diagnostic efficiencies in particular local systems of care (Figure 3)^33–39^.

In the United States, primarily for patients who present to an emergency department (ED), the swiftest path remains immediate hospitalization. This approach usually allows a quicker diagnostic evaluation and subsequent therapeutic intervention. Since much of the post-TIA stroke risk is front-loaded with stroke occurrence being greater in the first few days following a TIA, patients can be more closely monitored for potential neurologic deterioration, pending initiation of definitive stroke risk reduction therapies (Figure 3). Nevertheless, there are many patients with TIA who present to emergency, urgent care, or ambulatory settings many days or weeks after symptom onset when the risk of stroke, though still substantial, has declined and hospitalization is difficult to justify.

The “TIA clinic” as a systematized ambulatory urgent care paradigm may serve as an alternative to hospitalization. However, such an approach necessitates a high measure of preparatory organization, operational efficiency, and access to care with an appropriate commitment of resources. Successful TIA clinics have the attributes of fast-track access (i.e., referral of patients with TIA results for same-day or next-day appointments)^19,40–43^, expert assessment (i.e., by a vascular neurologist)^44,45^, and rapid access to appropriate diagnostic tests^46,47^, with management protocols using well-defined therapeutic workflows^48–53^ (Figure 3).

### Components of the transient ischemic attack evaluation

The comprehensive evaluation of patients with TIA begins with consideration of the three domains of Virchow’s triad and continues along a path that ideally will lead to identification of the causative mechanism of the index event (Figure 2). Regarding the final etiopathogenic adjudication of TIA, we favor the use of the Trial of ORG-10172 in Acute Stroke Treatment (TOAST) stroke classification paradigm (Table 1)^54^. The relative frequency of the various subtypes varies according to the populations studied and the methods used for assessment^55,56^. In this context, prioritizing imaging of the cerebral arterial system and identification of potential cardiogenic sources of embolization can be complemented by focused evaluation of additional etiologic considerations (e.g., thrombophilias) (Figure 2)^57–61^.

**Table 1.** Components of the TOAST classification for ischemic stroke causation.

TOAST ischemic stroke subtype
**Atherothromboembolic**
• Extracranial
• Intracranial
**Cardioembolic**
• Higher-risk (examples below)
◦ Atrial cardiopathy — atrial fibrillation
◦ Mitral stenosis with atrial fibrillation
◦ Mechanical prosthetic valves
◦ Severe left ventricular dysfunction
◦ Infective endocarditis
◦ Myxoma
• Lower risk (examples below)
◦ Mitral valve prolapse and annulus calcification
◦ Papillary fibroelastoma
◦ Lambl’s excrescences
**Lacunar infarction (i.e., hypertensive arteriolopathy)**
**Other determined etiology (examples below)**
• Non-atherosclerotic arteriopathies
• Neurovascular blunt trauma — dissection
• Paradoxical brain embolism
• Thrombophilias — congenital and acquired
**Unknown etiology**
• Type I: Incompletely evaluated
• Type II: Two or more potential causes (“double jeopardy”)
• Type III: Negative complete evaluation

We have made minimal stylistic modifications to the originally published classification for the sake of practical utilization. TOAST, Trial of ORG-10172 in Acute Stroke Treatment.

The cerebral vessels are imaged non-invasively with computed tomography angiography (CTA), magnetic resonance angiography (MRA), or neurovascular ultrasound (i.e., extracranial carotid/vertebral color Doppler and transcranial Doppler ultrasound). Modality choice depends on the clinical scenario, the underlying suspected vascular pathology, and the availability of particular imaging resources. MRA can be completed concurrently with MRI, which is very helpful in differentiating syndromes within the continuum of cerebrovascular syndromes (Figure 1)^47,58–61^. However, the MRA image is flow-dependent, and arterial anatomy must be inferred based on how arterial flow patterns (i.e., laminar and turbulent) affect the MRA appearance of the passage of protons in blood within the high-field magnets. Unfortunately, not all patients can undergo MRI studies, and access to this equipment may be difficult within the ED environment. On the other hand, CTA is more readily available in the ED setting^62^, and contemporary stroke urgent imaging protocols often incorporate CT perfusion imaging that, in the context of TIA evaluation (and by analogy to myocardial imaging), can help identify areas of reversible cerebral ischemia^63–66^. Extracranial (i.e., carotid and vertebral color) and intracranial (i.e., transcranial) Doppler provide more limited anatomic cerebrovascular information but are useful in those patients who cannot undergo MRA or CTA or when complementary information (e.g., vessel wall morphology, arterial flow dynamics, microembolic potential, and right-to-left shunts) is needed^67–69^. Finally, catheter cerebral angiography, though no longer a first-line tool in evaluation of patients with TIA, remains a very important tool in specific scenarios, such as (a) defining pathologic entities whose nature may render them inaccessible to the resolution of non-invasive vascular imaging (e.g., vasculitides), (b) qualifying patients for therapeutic intervention (e.g., assessing the degree of lumen stenosis as it compares with published normative references), and/or (c) providing a feasibility dimension for choosing a therapeutic approach (e.g., endovascular versus surgical)^70^.

The evaluation of potential cardiac causes for TIA begins with a basic 12-lead electrocardiogram, which may reveal overt rhythm abnormalities (e.g., previously undiagnosed atrial fibrillation) as well as other markers of stroke risk (e.g., P-wave changes of left atrial enlargement [LAE] or left ventricular hypertrophy). At present, comprehensive cardiac imaging can be accomplished by transthoracic (TTE) or transesophageal (TEE) echocardiography or both (Table 2). These two complementary approaches to ultrasonic cardiac imaging provide both morphologic and functional information about cardiac performance, uncovering abnormalities that must be factored in during risk stratification (Table 2), and TTE favors the evaluation of left ventricular contractility, mitral valve performance, left atrial volume, and the possible presence of right-to-left shunts^71,72^. By contrast, TEE offers increased resolution and sensitivity for left atrial pathology, including the left atrial appendage (LAA) and the interatrial septum; TEE also can provide information about the aortic arch and the potential presence of complex atherosclerotic plaques as a source of artery-to-artery cerebral embolism (Table 2). Cardiac CT or MRI may also complement or replace ultrasound-based cardiac assessment in the future, at least in selected populations. Incorporation of this cardiac imaging data into the patient’s risk stratification will facilitate the development of a management plan tailored to the specific patient^71,72^. Additionally, prolonged cardiac monitoring for intervals longer than 30 days, particularly using implantable loop recorders, has increased the detection of paroxysmal arrhythmias, particularly occult atrial fibrillation^73–76^. Thus, it is reasonable to incorporate long-term cardiac monitoring in the evaluation of patients with TIA suspected of having had an embolism, especially those with stigmata associated with atrial fibrillation (e.g., LAE).

**Table 2.** Complementary diagnostic strengths of transthoracic and transesophageal echocardiogram.

Finding	Comparison	Difference
**Left atrium and left atrial appendage**		
Left atrial enlargement	TEE =/> TTE	Moderate
Left atrial thrombus	TEE > TTE	Major
Spontaneous echo contrast	TEE > TTE	Major
Left atrial appendage thrombus	TEE > TTE	Major
Left atrial appendage dysfunction	TEE > TTE	Major
**Mitral and aortic valves**		
Stenosis	TEE =/> TTE	Minimal
Prolapse	TEE =/> TTE	Minimal
Masses	TEE > TTE	Moderate
**Interatrial septum**		
Patent foramen ovale	TEE > TTE	Moderate
Atrial septal aneurysm	TEE > TTE	Moderate
Lipomatous hypertrophy	TEE > TTE	Major
**Additional findings**		
Left ventricle anatomy and function	TTE > TEE	Minimal
Left ventricular apex function	TTE > TEE	Major
Aortic atheroma	TEE > TTE	Major
Transpulmonary right-to-left shunt	TEE > TTE	Major

TEE, transesophageal echocardiogram; TTE, transthoracic echocardiogram.

Finally, the evaluation of the hematologic domain for potential causes of TIA and ischemic stroke encompasses the identification of common stroke risk markers (e.g., diabetes and dyslipidemia) as well as any potential cause of an undiagnosed thrombophilia. Information obtained as part of the patient’s history (e.g., history of cancer, especially adenocarcinomas, unprovoked deep venous thrombosis, or autoimmune conditions) and the results of initial routine laboratory studies (e.g., hematocrit, platelet count, and prothrombin time) may indicate the need for more in-depth evaluation of hemostasis. The latter would lead to more specific testing of coagulation (e.g., thromboelastography), immunologic markers (e.g., antiphospholipid antibodies), and/or genetic testing (e.g., factor V Leiden mutation)^77^.

## Stroke risk stratification

The etiologic diagnosis of a presumptive TIA can lead to a stroke prevention strategy with accompanying risk reduction of long-term morbidity and mortality. The degree of stroke post-TIA risk varies by patient characteristics and etiopathogenic mechanism. The addition of diagnostic testing information improves the risk quantification in scoring systems. For example, the addition of a DWI-positive finding to the ABCD^2^ score yielded a different 90-day stroke risk (i.e., 0% for scores below 4 and up to 32.1% for scores of 7–9)^78,79^. The additional incorporation of variables about carotid artery stenosis in the ABCD^3^-I provided additional perspective about stroke risk at 7, 28, and 90 days^80^. A different scale incorporated information about atrial fibrillation with additional predictive value for post-TIA stroke risk prediction^81^. Subsequently, the ABCDE⊕ score described enhanced predictability of subsequent cerebral ischemic events by adding “etiology” and “DWI positivity” to the ABCD score^82^, and a more complex comprehensive stroke recurrence model (i.e., incorporating demographic, clinical, and radiological findings) to patients with TIA, particularly those with DWI abnormalities, has also been validated^83,84^. All of these systems highlight the necessity of a detailed evaluation of patients with TIA, not different from the evaluation of patients with ischemic stroke, to define the best therapies for stroke risk reduction tailored to individual patients^85–88^.

Some general principles about stroke risk stratification may assist with specific assessments: (a) irrespective of the underlying vascular pathology, the degree of flow reduction (i.e., stenosis) is proportional to the stroke risk^89^; (b) irrespective of the underlying cardiac pathology, the degree of forward flow impairment (e.g., LAE, left ventricular dysfunction, and mitral stenosis) is proportional to the stroke risk^90^; and (c) stroke risk is additive, increasing in the presence of multiple causative stroke mechanisms (i.e., “double jeopardy”)^91^.

## Tailored management plan

### General measures

Some general care measures include isotonic crystalloid solutions that can be easily administered to patients with TIA as soon as they arrive in the ED. It is an inexpensive and low-risk step in line with guidelines for the early management of acute ischemic stroke. In addition, it addresses the fact that about 50% of patients with stroke present dehydrated. Along the same lines, any patient with TIA should receive oxygen supplementation to keep an oxygen saturation above 92%, careful blood pressure (BP) control, and serum glucose regulation^92–95^.

### Target-specific measures

***Antiplatelet therapy.*** Early antithrombotic therapy leads to an about 80% relative reduction of stroke risk in patients with TIA^94,95^. Aspirin, at doses of 50 to 325 mg per day, has been consistently found to reduce the odds of a subsequent stroke in patients with previous TIA, and the published relative risk reduction rates vary between 13 and 70% depending on the subpopulation being studied and the specified outcomes^96–98^. The benefit is highest when given early in the time course following TIA^99^. Clopidogrel monotherapy has not been studied in patients with TIA, and ticagrelor monotherapy appears to be 30% more effective than, and as safe as, aspirin in reducing major adverse events (i.e., stroke, myocardial infarction, or death) but only in patients with TIA associated with an ipsilateral stenotic lesion^100,101^. Finally, cilostazol has been shown in Asian studies to be superior to aspirin in reducing vascular events in patients with stroke, a finding that may translate to patients with TIA in the context of the cerebrovascular continuum (Figure 1)^102,103^.

Dual antiplatelet therapy is also an important therapeutic consideration in the management of patients with TIA. The combination of aspirin plus clopidogrel has been found to be superior to aspirin alone in reducing stroke risk when used for hyperacute treatment of patients with TIA and to have insignificant additional hemorrhagic risks^104–106^. The combination of ticagrelor and low-dose aspirin in patients with minor stroke or TIA has been found to be associated with about 20% relative stroke risk reduction, although it carried an increased risk of severe bleeding^107^. The combination of aspirin plus dipyridamole is not superior to clopidogrel alone in reducing the risk of stroke^108^. Cilostazol combined with clopidogrel or aspirin results in improved stroke risk reduction with a similar risk of severe or life-threatening bleeding compared with either agent alone^102,109,110^. However, “triple therapy” using aspirin, clopidogrel, and dipyridamole not only failed to show improved stroke risk reduction but conveyed an increased risk of major bleeding^111^.

***Anticoagulation.*** Vitamin K antagonists, particularly warfarin, have been used for many years to reduce the risk of thromboembolism risks. In the context of ischemic stroke prevention, the main application of warfarin has been in patients with atrial fibrillation, mechanical heart valve prostheses, and other cardioembolic etiologies^88,95^. In patients with “non-valvular” atrial fibrillation (i.e., without concomitant mitral stenosis), warfarin confers a relative risk reduction for stroke of about 60 to 70% in comparison with no antithrombotic treatment being administered and by 30 to 40% when compared with treatment with antiplatelet agents^112^.

Two other classes of oral anticoagulants for the treatment of patients with non-valvular atrial fibrillation are currently available: (a) direct thrombin inhibitors (i.e., dabigatran) and (b) factor Xa inhibitors (i.e., apixaban, rivaroxaban, and edoxaban). These direct oral anticoagulants (DOACs) are at least of comparable effectiveness to warfarin in patients with non-valvular atrial fibrillation with fewer major complications^113–122^. The appeal of the DOACs is that they had shorter half-lives, had fewer bleeding complications, were thought to have fewer food and drug interactions, and required no laboratory monitoring. However, DOACs are now known to have significant drug interactions, particularly in the presence of other substances whose metabolism includes the P glycoprotein pathway^123^. In this context, monitoring of DOACs anticoagulant effect using either anti-factor Xa levels^124,125^ or serum levels^126,127^ is an ongoing area of development. The role of monitoring may also be important in patients with chronic renal insufficiency, as DOACs are variably excreted by the kidney and their use can be complex in these patients^118^.

**Figure 4.**
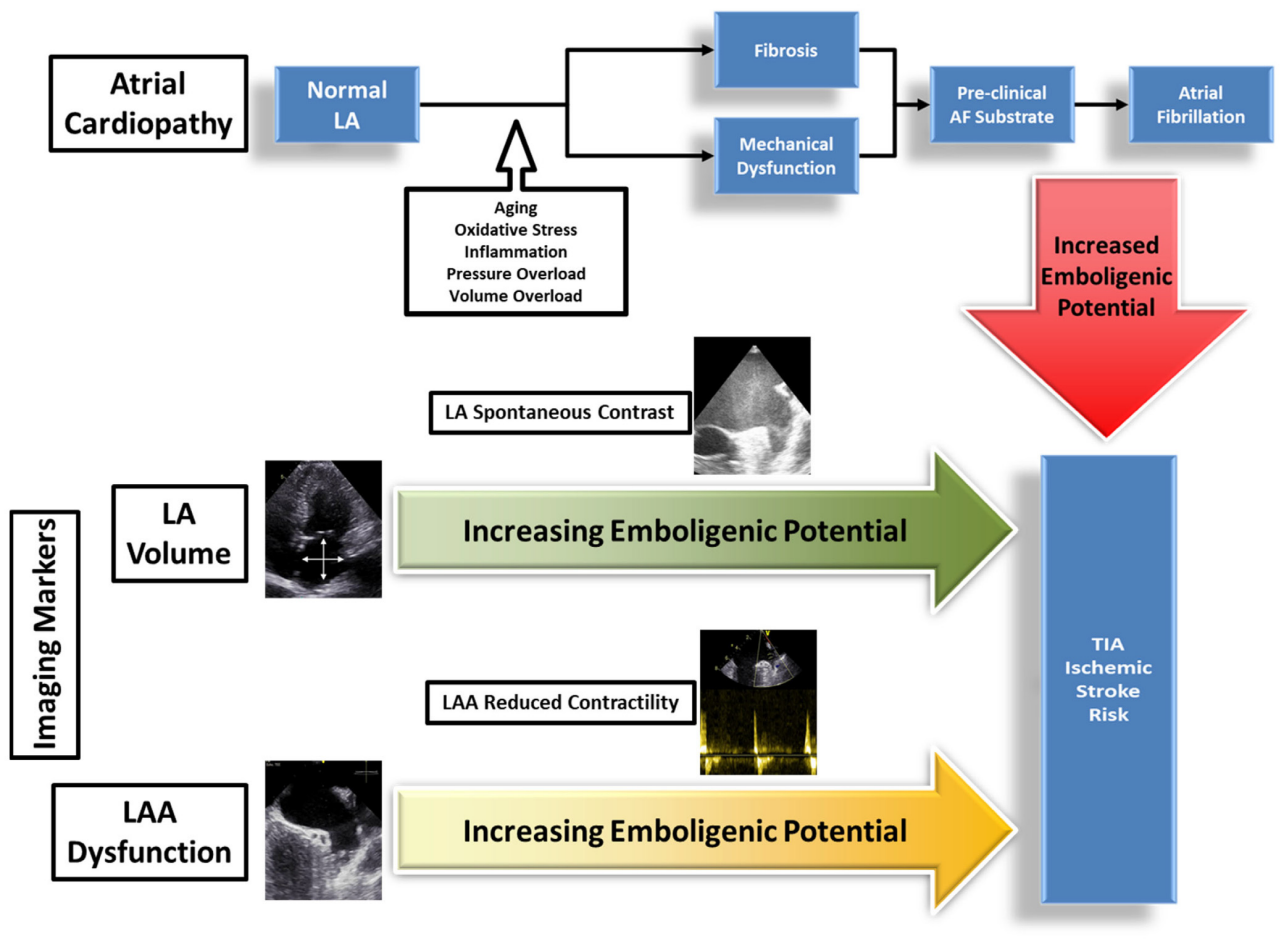
Atrial cardiopathy and its impact on atrial fibrillation. A normal left atrium can undergo progressive fibrosis and mechanical dysfunction due to the negative influence of multiple factors, eventually leading to the development of atrial fibrillation. Such a pathologic process can be identified, quantified, and monitored using echocardiographic testing geared at assessing left atrial and left atrial appendage morphology and function. AF, atrial fibrillation; LA, left atrium; LAA, left atrial appendage; TIA, transient ischemic attack.

Owing to its frequency, importance, evolving concepts, and response to anticoagulation, additional attention to atrial fibrillation is warranted. This prevalent arrhythmia does not occur in isolation or by happenstance and is more likely a component of a condition known as *left atrial cardiopathy* (Figure 4)^75,76,128,129^. This entity carries with it a pathologic substrate that includes progressive atrial fibrosis, LAE, lack of contractility, aberrant electrical conduction, and LAA dysfunction^130,131^. All of these findings appear to be stigmata for increased risk of atrial fibrillation, even in patients in whom an arrhythmia has not yet been identified. Therefore, it may be reasonable for clinicians to consider anticoagulation in selected patients with TIA with these findings, for whom there is a very high suspicion for unrecognized atrial fibrillation^128,132–134^.

In this context, during the last decade, the decision of whether to anticoagulate a patient with TIA with atrial fibrillation has been oversimplified by reducing it to comparing the “stroke risk” scored using a predetermined system such as the CHA_2_DS_2_-VASc (i.e., congestive heart failure, hypertension, age, diabetes, previous stroke, vascular disease, age, and sex category) with the “hemorrhagic risk” calculated using a system such as HAS-BLED (hypertension, abnormal renal and liver function, stroke, bleeding, labile international normalized ratios, elderly, and drugs or alcohol). Unfortunately, such arithmetic comparisons do not provide an accurate assessment that can be reliably used in any one specific patient^135,136^. The CHA_2_DS_2_-VASc score suffers from a limitation common to previously described scoring systems: other than congestive heart failure, its remaining components do not represent stroke risk which is *directly attributable* to cardiogenic embolism and which anticoagulation could be expected to modify. On the other hand, the limitations of the HAS-BLED score are more operational (Table 3). Using the intended definitions of the components renders most of them modifiable, arbitrary, or prone to incorrect scoring, making any scoring result suspect or at least not absolute (Table 3). In addition, HAS-BLED cannot be used to calculate the hemorrhagic risk of patients treated with DOACs.

**Table 3.** Limitations of the use of the HAS-BLED score.

Item	Definition	Limitations
Hypertension	“Uncontrolled hypertension >160 mm Hg systolic”	Modifiable by blood pressure control, with consequent score reduction
Abnormal renal/ liver function	“Chronic dialysis, renal transplantation, or serum creatinine (2.26 mg/dL)” & “Chronic hepatic disease (e.g., cirrhosis) or biochemical derangement (e.g., bilirubin >2x AND AST/ALT/AP >3x Upper Limits of Normal”	Potentially modifiable, with consequent score reduction
Stroke	“Previous history, particularly lacunar”	Unmodifiable
Bleeding history or predisposition	“Prior Major Bleeding (i.e., any bleeding requiring hospitalization, reducing hemoglobin by > 2 g/L, or transfusion but NOT Hemorrhagic Stroke) or Anemia”	Commonly scored incorrectly based on prior hemorrhagic stroke
Labile international normalized ratios	“Therapeutic time in range <60%”	Modifiable, with consequent score reduction, and not applicable to direct oral anticoagulants
Elderly	“>65 years”	Arbitrary
Drugs / Alcohol	“Antiplatelet agents or NSAIDs” or “≥8 Units ETOH consumption per week” or both	Modifiable, with consequent score reduction

AST/ALT/AP, aspartate aminotransferase / alanine aminotransferase / alkaline phosphatase; ETOH, ethanol alcohol; HAS-BLED,hypertension, abnormal renal and liver function, stroke, bleeding, labile international normalized ratios, elderly, and drugs or alcohol; NSAID, non-steroidal anti-inflammatory drug.

***Arterial revascularization.*** Patients with TIA etiologically associated with an ipsilateral extracranial carotid atherosclerotic lesion causing more than 50% diameter reduction (i.e., stenosis) should be evaluated for possible revascularization, either by carotid endarterectomy or by carotid artery stenting^137–140^. The procedure should be carried out preferably within two weeks of the index event and ideally as soon as possible following the index TIA event (<48 hours). Revascularization should be carried out at high-volume procedure centers, where the risk of major complication in this patient population is monitored and is about 6% or less^141–144^.

Following a TIA in the vertebrobasilar system, elective stenting of a relevant extracranial vertebral artery for stroke prevention may be considered in individuals with one or more of the following characteristics: (a) patients who remain symptomatic despite optimal medical therapy, including risk factor modification and antithrombotic agents, (b) patients with recurrent symptomatic unilateral high-grade stenosis of a dominant vertebral artery, (c) patients with recurrent symptomatic bilateral high-grade stenosis, giving preferential treatment to the dominant vertebral artery, and (d) patients with a high-grade stenosis in an isolated (i.e., no contralateral collateral support) vertebral artery and recurrent symptoms referable to ischemia of the ipsilateral posterior inferior cerebellar artery^145–147^. Data for vertebral artery stenting are not as voluminous as those for carotid stenting. However, the most recent guidelines emphasize the primacy of medical therapy and the need for ongoing clinical trials^148^.

In patients with intracranial atherosclerotic lesions, the application of endovascular techniques (i.e., angioplasty and stenting) is a complex subject, particularly because patient selection and operator experience are critical factors in determining the feasibility and reasonableness of procedural application. The current recommendations are to manage these patients with “maximal medical therapy”^95,146^. Nevertheless, it is reasonable to consult expert operators, particularly if these patients remain symptomatic with multiple recurrent TIAs despite conservative management^148^.

***Paradoxical brain embolism.*** Stroke prevention in patients whose TIA is the result of a thromboembolic process that originates in the venous system and reaches the brain via a right-to-left shunt has become a subject of increased complexity and warrants careful consideration. Clinicians should suspect paradoxical embolism in specific scenarios, such as long-distance air and land trips, cerebrovascular syndromes presenting with concomitant acute pulmonary embolism or deep venous thrombosis, concomitant obstructive sleep apnea (OSA) in the risk profile, or known right-to-left shunts^149–153^.

**Figure 5.**
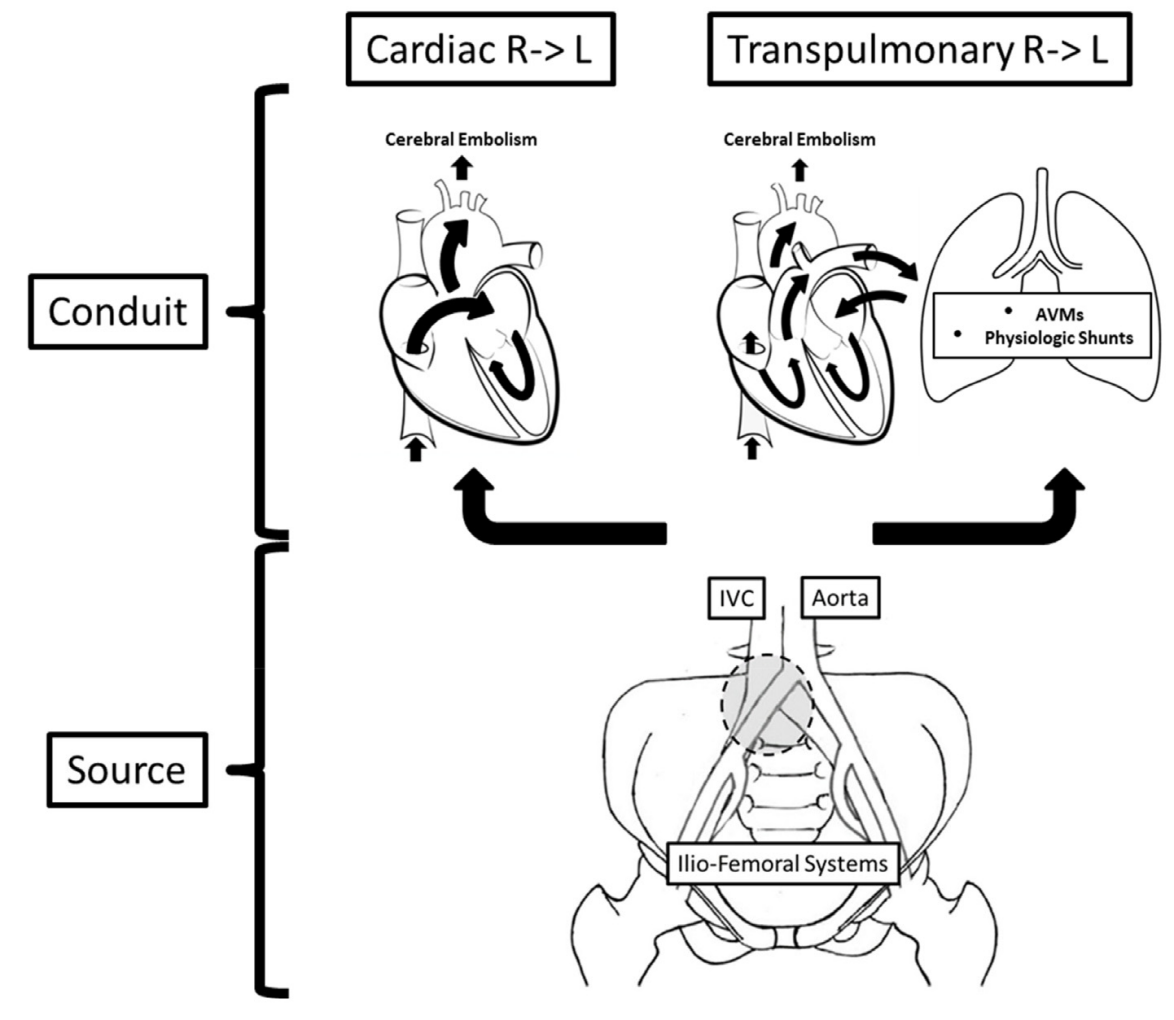
Components of the pathogenic mechanism of paradoxical brain embolism. Two major conduits allow venous-originated emboli to reach the cerebral circulation: cardiac and transpulmonary. The source of embolism, on the other hand, can be located anywhere in the venous structures of the pelvis and lower extremities. In this regard, the gray circle points to the typical location of May-Thurner iliac arteriovenous anomalies, an important risk factor for thromboembolism. See text for expanded discussion. AVMs, arteriovenous malformations; IVC, inferior vena cava; R -> L, right-to-left.

The basis of the assessment of paradoxical brain embolism begins with the understanding of the two operating components of this pathogenic mechanism: (a) a thromboembolic *source* and (b) an embolic *conduit* with an associated right-to-left shunt (Figure 5). The latter is frequently transcardiac, but the role of transpulmonary right-to-left shunting continues to gain increasing importance. In this context, although the bulk of the existing literature on paradoxical brain embolism is centered on the diagnosis and management of patent foramen ovale (PFO), this appears to be a seriously narrow perspective, obviating many other details that should influence how these patients are evaluated and managed and that warrant additional consideration in the context of our discussion.

**Figure 6.**
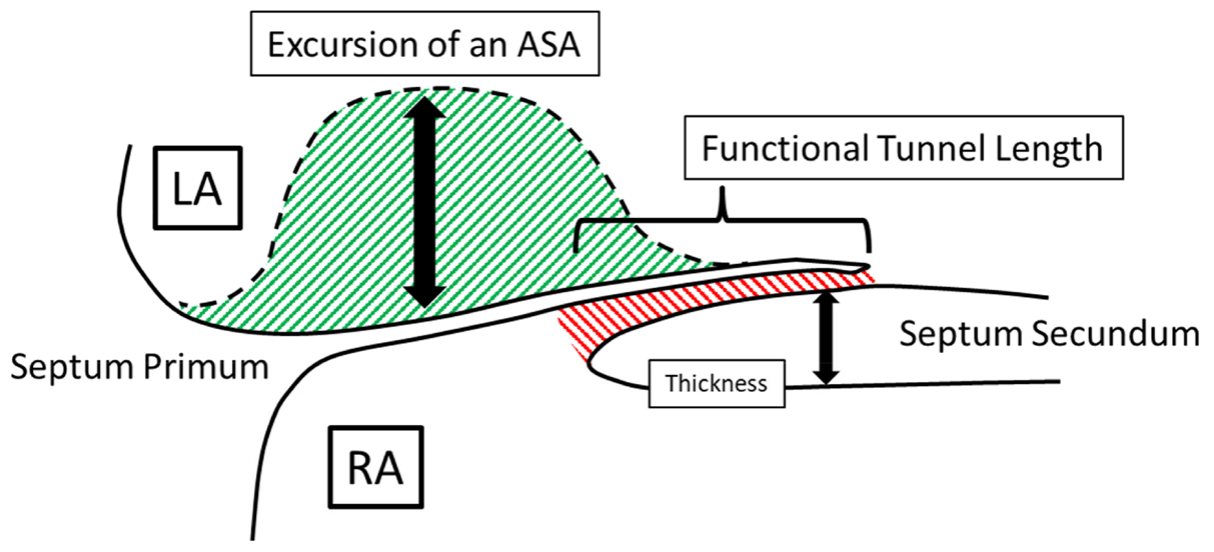
Morphologic attributes of a patent foramen ovale that contribute to its complexity. The red striped area designates the tunnel between the septum primum on the left atrial side and the septum secundum on the right atrial side. The green striped area demonstrates the potential excursion and mobility of an atrial septal aneurysm. The thickness of the septum secundum has also been associated with increased complexity. See text for expanded discussion. ASA, atrial septal aneurysm; LA, left atrium; RA, right atrium.

The prevalence of PFO in the general population is about 25 to 30%, and the literature supports an association with an increased risk of ischemic stroke^154^. However, although the basic anatomic defect resulting in a PFO is the failure of the caudal portion of the septum primum and the cranial portion of the septum secundum to fuse after birth, this defect is anything but uniform, in both morphology and function. This fact greatly complicates our interpretation of the existing literature addressing its significance and best management approach^155^. In principle, a PFO is not a “hole in the heart” as it is commonly referred to when discussing findings with patients. It is more like a “tunnel” with a “slit valve” that allows the passage of blood and embolic particles between the atria (Figure 6). However, even this is an oversimplification, and the complexity of PFOs increases with the presence of one or more of the following: (a) long tunnel length (≥8–10 mm), (b) multiple fenestrations into the left atrium, (c) atrial septal aneurysm, (d) large opening, (e) shunt at rest, (f) hybrid defect, (g) thick septum secundum (≥ 10 mm), (h) Eustachian valve or Chiari network, or (i) an angle between the PFO and the inferior vena cava of not more than 10 degrees^155^. Taking these concepts into consideration, it is easy to understand and predict that no one treatment strategy is bound to have uniform effectiveness in stroke risk reduction in this subpopulation of individuals. Indeed, this may explain why the effectiveness of one or another antithrombotic regimen has a variable effect on stroke prevention^156,157^. Such heterogeneity is also likely to be the reason why, of the six most important published randomized clinical trials, two showed no benefit of percutaneous closure, two showed equivocal results, and only the most recent two showed some benefit, although they selectively included patients with more complex types of PFO, in fact, considered “high risk” (Table 4)^158^. At present, it seems reasonable to consider percutaneous PFO closure in carefully selected patients, with clearly selected goals of treatment (e.g., patients who frequently engage in activities that promote right-to-left shunting, such as SCUBA [i.e., self-contained underwater breathing apparatus] diving)^159^, particularly because the procedure has been associated with potentially significant complications, including about 1% risk of thromboembolism, 1% risk of pericardial tamponade, 3 to 5% risk of residual shunt, 1% risk of device fracture, and, most importantly, up to 5% risk of atrial fibrillation. The last of these is important because no one would be interested in replacing one cause of ischemic stroke for another, especially if it means that the patient will have to remain anticoagulated despite the procedure. However, an additional factor when considering percutaneous PFO closure is that patients with concurrent PFO and ASA often display significant left atrial dysfunction, simulating atrial fibrillation, and that PFO closure may be beneficial by normalizing left atrial function^160^.

**Table 4.** Randomized prospective clinical trials of percutaneous patent foramen ovale (PFO) closure.

Parameter	Closure-I (No Benefit)	PC trial (No Benefit)	Respect (Inconclusive)	Reduce (Inconclusive)	Close (Benefit)	Defense-PFO (Benefit)
**Patients, number**	909	414	980	664	663	120
**Mean age, years**	46	44.5	46	42.2	43.3	51.8
**Moderate-large R->L, %**	53	65.6	48.8	81.3	100	53
**ASA, %**	36.6	23.7	35.7	20.6	32.8	10
**Antithrombotic Rx**	Aspirin, OAC	APT, OAC	APT, OAC	APT	APT, OAC	APT
**OAC, %**	34	31	25	0	28	0
**Follow-up, months**	44	49	70.8	38.4	63.6	24
**Stroke, % - M**	3.1	2.4	5.8	5.4	6.0	10.5
**Stroke, % - C**	2.9	0.5	3.6	1.4	0.0	0
**TIA, % - M**	4.1	3.3	4.8	–	–	2.0
**TIA, % - C**	3.1	2.5	3.4	–	–	0
**Death, % - M**	0	0	2.2	0	0	0
**Death, % - C**	0	1.0	1.4	0.5	0	0

The stroke prevention outcomes are highlighted for comparison, indicating whether or not their results showed benefit of closure, or if they were inconclusive. See text for additional details. APT, antiplatelet therapy; ASA, atrial septal aneurysm; C, closure arm; M, medical arm; OAC, oral anticoagulation; R->L, right-to-left shunt; Rx, regimen; TIA, transient ischemic attack.

In assessing the best management strategy for patients with paradoxical brain embolism, one should consider both the transpulmonary conduits for paradoxical embolism and the source of the thromboembolic process (Figure 5). The two important underlying substrates for transpulmonary right-to-left shunting are (a) pulmonary arteriovenous malformations (AVMs) and (b) pulmonary physiologic and reactive shunts^159^. Pulmonary AVMs have a prevalence rate of about 1 per 2,600, more than 80% of patients have hereditary hemorrhagic telangiectasia, and nearly 95% of them first present with an ischemic stroke due to paradoxical embolism^161^. Interestingly, a potentially modifiable factor identified in these patients is iron deficiency, shown to mediate a serotonergic pro-aggregatory platelet response facilitated by a dysfunctional platelet monoamine oxidase^162^. These anomalies can be readily diagnosed using chest CTA and may require treatment by percutaneous embolization^163^. Perhaps more frequently, transpulmonary right-to-left shunting can result from physiologic pulmonary shunts that have been known to reactively open in response to a variety of stimuli such as exercise workload, hypoxemia, catecholamine infusions, and excessive pulmonary embolization^159^. The presence of transpulmonary shunting can be identified by TEE but generally not by TTE since microbubbles from agitated intravenous saline can be reliably visualized passing via the pulmonary veins using only the former.

**Figure 7.**
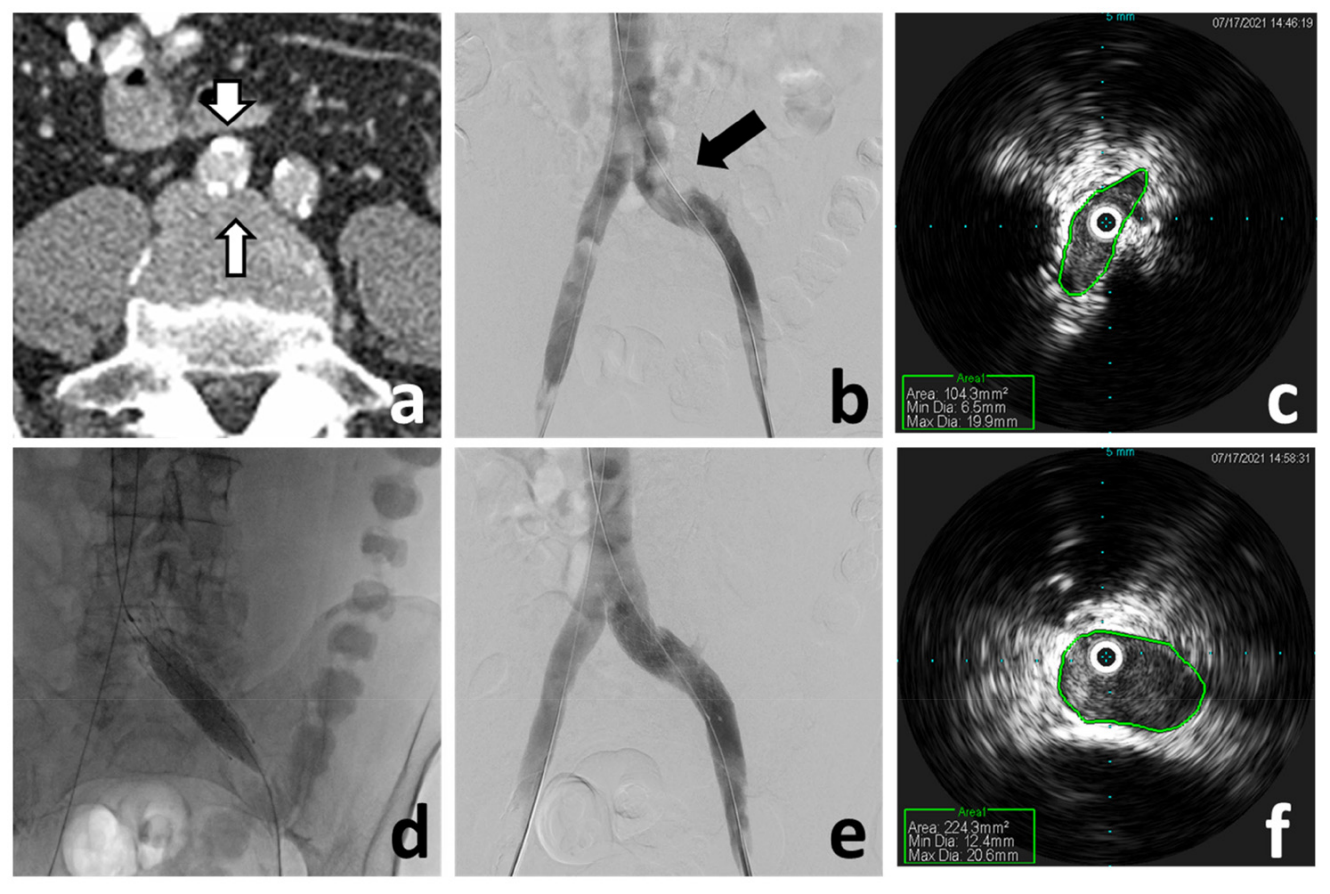
May–Thurner anomaly diagnosis and endovascular treatment. (**a**) Abdomen-pelvis computed tomography angiography demonstrates compression of the left common iliac vein (narrow arrow) by a calcific right common iliac artery (wide arrow). (**b**) Venography demonstrates the external compression of the left common iliac vein (arrow), although it does not accurately represent the severity. (**c**) Intravascular ultrasound (IVUS) of the left common iliac vein demonstrates the significant lumen reduction (area = 104.3 mm^2^). (**d**) Balloon post-dilatation of the deployed left iliac vein stent. Venography (**e**) and IVUS (**f**) post-stenting demonstrate correction of the external compression and stenosis (area = 224.3 mm^2^).

Regarding the source, the venous system of the pelvis and lower limbs requires a thorough evaluation in patients suspected of having experienced a paradoxical brain embolism^164^. It makes no clinical sense to therapeutically address the conduit, such as by closing a PFO or embolizing a pulmonary AVM, and ignore a venous source of thromboembolism, particularly since the pulmonary circulation would continue to be at risk despite the right-to-left shunt having been closed. In this context, not finding imaging evidence of a deep venous thrombosis, in either the pelvis or the lower limb veins, should not be considered unequivocal proof that there was not one. In fact, a more important and frequent relevant finding is that of an abnormality of the venous anatomy, one associated with increased risk of venous thromboembolism. The prototype of this finding is the May-Thurner anomaly: external compression of the left common iliac vein by the right common iliac artery which, over time, causes progressive intraluminal fibrosis, stenosis, and stagnation of the venous return, all potentially leading to venous thromboembolism (Figure 5 and Figure 7)^164^. Identification of this anomaly, which may otherwise be asymptomatic, carries the additional significance in that percutaneous correction of the venous defect is both feasible and effective (Figure 7)^165–167^.

Finally, no discussion about paradoxical brain embolism is complete without addressing the role that OSA plays in the pathogenesis and risk of stroke. The literature underscores the fact that, even though OSA has been found to be an independent risk factor for stroke, many patients with TIA and stroke never undergo polysomnography as part of their evaluation. This is troublesome since continuous positive airway pressure may be beneficial in reducing stroke risk^168^. In addition to the fact that cardiac arrhythmias, particularly atrial fibrillation, are more common in individuals with OSA, the physiologic derangements produced by episodes of obstructive apnea during sleep have a profound effect on patients with right-to-left shunting^169^. In particular, the inspiratory effort against the obstructed airway (i.e., Mueller maneuver) leads to intrathoracic pressure changes that mirror the Valsalva maneuver while resembling its *release*: a pronounced reduction of the intrathoracic pressure^169^. In turn, this leads to reduction of the right atrial pressure and an increase in any right-to-left shunt^169^. Thus, it is easy to see that a key component of the management of patients with paradoxical brain embolism is the identification and treatment of concomitant OSA.

### Vascular risk factor modification

There is an increased risk of cardiovascular events following TIA or stroke, particularly in patients with extensive atherosclerosis. In this context, arterial hypertension, diabetes, dyslipidemia, and smoking are the leading modifiable atherogenic risk factors for cerebrovascular and coronary insufficiency^94^. It is essential that clinicians address these modifiable factors with an emphasis on important core health behaviors (i.e., smoking cessation, physical activity, proper dietary patterns, and weight control) and monitoring health-related metrics (i.e., lipid profiles, BP, and glucose control)^170,171^. Along these lines, BP control (i.e., <140/90 mm Hg in most patients and <130/80 mm Hg in diabetics and ideally normotension [120/80 mm Hg]) for long-term prevention of recurrent events is of paramount importance for stroke risk reduction^94^. Intensive lipid-lowering therapy with statins should be instituted in patients with atherosclerosis and low-density lipoprotein cholesterol (LDL-C) greater than 100 mg/dL^172,173^. The proprotein convertase subtilisin kexin 9 (PCSK9) inhibitors (e.g., alirocumab and evolocumab) have also demonstrated significant LDL-C lowering and risk reduction in coronary heart disease for statin-intolerant patients^174,175^, although the specific benefit for patients with cerebrovascular disease is uncertain^176^.

Those patients with TIA at risk for metabolic syndromes should undergo measurements of hemoglobin A1c (HbA1C) and body mass index^94^. Patients at risk should be treated with a program that includes exercise, diet, weight reduction, and glucose-lowering drugs, with a goal of reducing serum glucose fasting values to less than 126 mg/dL^94,177,178^. Data from 2017 suggest that hyperglycemia may have a particularly negative effect on patients treated with clopidogrel for whom glycemic control may be particularly essential^179^.

## Conclusions

A rapid diagnosis offers the opportunity for early post-TIA stroke risk reduction by defining the etiopathogenesis of the acute cerebrovascular event. In the future, better clinical risk stratification prediction systems with diagnostic tools and biomarkers for stroke risk identification coupled with new therapeutic modalities for stroke risk reduction may support enhanced systems of care for patients with TIA without over-reliance on hospitalization.
